# A fully human anti-Ep-CAM scFv-beta-glucuronidase fusion protein for selective chemotherapy with a glucuronide prodrug

**DOI:** 10.1038/sj.bjc.6600143

**Published:** 2002-03-04

**Authors:** M de Graaf, E Boven, D Oosterhoff, I H van der Meulen-Muileman, G A Huls, W R Gerritsen, H J Haisma, H M Pinedo

**Affiliations:** Department of Medical Oncology, Division of Gene Therapy, Vrije Universiteit Medical Centre, PO Box 7057, 1007 MB Amsterdam, The Netherlands; Department of Immunology, Utrecht Medical Centre, Utrecht, The Netherlands; Department of Therapeutic Gene Modulation, University Centre for Pharmacy, University of Groningen, PO Box 196, 9700 AD Groningen, The Netherlands

**Keywords:** anthracyclines, cancer chemotherapy, β-glucuronidase, glucuronide, human fusion protein

## Abstract

Monoclonal antibodies against tumour-associated antigens could be useful to deliver enzymes selectively to the site of a tumour for activation of a non-toxic prodrug. A completely human fusion protein may be advantageous for repeated administration, as host immune responses may be avoided. We have constructed a fusion protein consisting of a human single chain Fv antibody, C28, against the epithelial cell adhesion molecule and the human enzyme β-glucuronidase. The sequences encoding C28 and human enzyme β-glucuronidase were joined by a sequence encoding a flexible linker, and were preceded by the IgGκ signal sequence for secretion of the fusion protein. A CHO cell line was engineered to secrete C28-β-glucuronidase fusion protein. Antibody specificity and enzyme activity were retained in the secreted fusion protein that had an apparent molecular mass of 100 kDa under denaturing conditions. The fusion protein was able to convert a non-toxic prodrug of doxorubicin, *N*-[4-doxorubicin-*N*-carbonyl(oxymethyl)phenyl]-*O*-β-glucuronyl carbamate to doxorubicin, resulting in cytotoxicity. A bystander effect was demonstrated, as doxorubicin was detected in all cells after *N*-[4-doxorubicin-*N*-carbonyl(oxymethyl)phenyl]-*O*-β-glucuronyl carbamate administration when only 10% of the cells expressed the fusion protein. This is the first fully human and functional fusion protein consisting of an scFv against epithelial cell adhesion molecule and human enzyme β-glucuronidase for future use in tumour-specific activation of a non-toxic glucuronide prodrug.

*British Journal of Cancer* (2002) **86**, 811–818. DOI: 10.1038/sj/bjc/6600143 www.bjcancer.com

© 2002 Cancer Research UK

## 

Treatment of solid tumours with conventional cytotoxic drugs lacks selectivity and thus results in toxic dose-limiting side effects. In addition, the survival of drug-resistant tumour cells will occur due to insufficient drug concentrations at the site of the tumour. Selectivity in cancer therapy could be conveyed by monoclonal antibodies against tumour-associated antigens. These antibodies have been used as carriers of cytotoxic agents, such as conventional cytotoxic drugs, radionuclides, or toxins derived from plants or bacteria. Monoclonal antibodies can also be used for tumour-selective delivery of an enzyme that converts a relatively non-toxic prodrug into its toxic drug. This approach is called antibody-directed enzyme prodrug therapy (ADEPT) (Bagshawe, 1987; Senter *et al*, 1988). The idea of using antibodies to localize enzymes to the tumour was originally conceived by Philpott *et al* (1973). The selective activation of the prodrug at the site of the tumour should enhance the drug concentration in the tumour and result in a better anti-tumour effect and a reduction of systemic toxicity.

The occurrence of an immune response hampers repeated administration of a non-human enzyme immunoconjugate, as prolonged treatment may prove necessary for optimal efficacy in solid tumours. In the clinical trial reported by Sharma *et al* (1992) all patients developed detectable antibodies against the murine antibody fragment and against the bacterial enzyme within 10 days after a single dose of antibody-enzyme conjugate. Therefore, it is desirable to use human proteins, as is also underlined by the recent clinical progress with engineered human antibodies (McLaughlin *et al*, 1998; Pegram *et al*, 1998; Vincenti *et al*, 1998).

A suitable candidate for a human prodrug-converting enzyme could be human β-glucuronidase (GUSh). GUSh is localised intracellularly in microsomes and lysosomes and its activity is not detectable in human blood. This enzyme is able to convert a hydrophilic prodrug of doxorubicin, *N*-[4-doxorubicin-*N*-carbonyl (oxymethyl)phenyl]-*O*-β-glucuronyl carbamate (DOX-GA3), which is not able to cross cell membranes (Houba *et al*, 1996). Therefore, DOX-GA3 is not available for conversion by the endogenous enzyme under normal circumstances. An additional advantage of this human enzyme is the fact that, in larger tumours, the glucuronyl-doxorubicin prodrug has an inhibiting effect on tumour growth on its own (Bosslet *et al*, 1995; Houba *et al*, 2001a). The success of the glucuronyl-doxorubicin prodrug is ascribed to release of the enzyme from necrotic tumour cells and macrophages in larger tumours (Bosslet *et al*, 1998). A potential limitation of glucuronide prodrugs in monotherapy is that they are not activated homogeneously throughout the tumour tissue, but only at the site of necrosis and inflammatory cell infiltration. Furthermore, small tumour lesions do not yet contain necrotic areas. The efficacy of prodrug monotherapy could be increased by exogenous administration of targeted β-glucuronidase.

Previous studies with monoclonal antibodies against the pan-carcinoma antigen, epithelial cell adhesion molecule (Ep-CAM), have shown tumour selectivity (De Bree *et al*, 1994; Riethmuller *et al*, 1994). We have already demonstrated that chemical conjugates consisting of the anti-Ep-CAM monoclonal antibody 323/A3 and GUSh specifically localised into the Ep-CAM expressing tumour in nude mice bearing human ovarian cancer xenografts (Houba *et al*, 2001b). Administration of the conjugate and DOX-GA3 in this model resulted in an enhanced inhibition of tumour growth as compared to that obtained with DOX-GA3 alone. The production of chemical conjugates, however, is very laborious. The production of genetic fusion proteins is obviously an attractive alternative. We have previously shown that fully functional scFv-GUSh fusion proteins can be produced in mammalian cells (Haisma *et al*, 1998a,b). Thus far, the scFvs used were from murine origin. Genetic fusion of GUSh to a human scFv would result in a fully human, and as a result in a non-immunogenic, fusion protein.

In the present study, we constructed a fusion protein consisting of the genes encoding the human scFv against Ep-CAM (C28) and GUSh. The fusion protein was expressed in eukaryotic cells, because GUSh requires N-linked glycosylation for activity (Tabas and Kornfeld, 1980). The construct contained a signal sequence at the 5′ end for secretion and a myc- and 6His-tag at the 3′ end for easy detection and purification. The enzymatic activity, antibody-binding specificity, and prodrug activation of the secreted fusion protein were analysed. We demonstrate that mammalian cells transduced with the C28-GUSh construct secrete fully functional fusion protein. Therefore, this fully human construct is a candidate for tumour-specific activation of the prodrug DOX-GA3.

## MATERIALS AND METHODS

### Materials

Pwo DNA polymerase, PCR buffer and dNTPs were obtained from Roche Biochemicals (Almere, The Netherlands). Restriction enzymes were purchased from New England Biolabs (Beverly, MA, USA) and Life Technologies (Breda, The Netherlands). The kits used for DNA isolation, purification, and extraction from agarose gel, were from Qiagen (Hilden, Germany). The substrate 4-methylumbelliferyl-β-*D*-glucuronide trihydrate (4-MuGlu) substrate was purchased from Sigma-Aldrich (Zwijndrecht, The Netherlands).

Doxorubicin was derived from Pharmacia and Upjohn (Woerden, The Netherlands). The prodrug DOX-GA3 was synthesised as described (Leenders *et al*, 1999).

### Cell lines

The COS-7 and CHO cell lines were obtained from the American Type Culture Collection (Rockville, MD, USA) and were grown in Dulbecco's modified Eagle's medium (DMEM) supplemented with 5% heat-inactivated FCS (Life Technologies) and antibiotics in a humidified atmosphere containing 5% CO_2_ at 37°C. The human ovarian cancer cell line OVCAR-3 (Hamilton *et al*, 1983) was grown in DMEM supplemented with 10% FCS and antibiotics. The human glioma-derived cell line U118 MG (glioblastoma multiforme) was obtained from Dr JT Douglas (Gene Therapy Centre, University of Alabama at Birmingham, Birmingham, AL, USA) and grown under the same conditions.

### Construction of pC28-GUSh

The anti-Ep-CAM scFv C28 was derived from scFv UBS-54, which was isolated from a semi-synthetic phage antibody display library in a pHEN vector (Huls *et al*, 1999). An *Sfi*I/*Not*I fragment encoding the scFv C28 was isolated from the pHEN vector and cloned into the eukaryotic expression vector, pSTCF (Arafat *et al*, 2000), containing a secretion signal and a myc- and 6His-tag. The pSTCF vector was digested with the same enzymes. A flexible (Gly_4_Ser)_2_ linker was introduced downstream of C28. For this purpose, two overlapping primers with 5′ phosphorylation were designed (Table 1

**Table 1:**
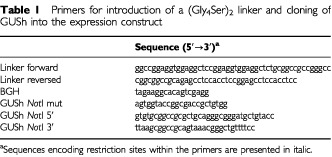
Primers for introduction of a (Gly_4_Ser)_2_ linker and cloning of GUSh into the expression construct

). Hybridization of the primers resulted in a 5′ end that was compatible with *Not*I and a 3′ end compatible with *Apa*I. The primers were designed in such a way that the *Not*I site at the 5′ end of the linker disappeared upon ligation into the vector. A new *Not*I site was introduced at the 3′ end of the sequence encoding the linker to allow insertion of GUSh downstream of the (Gly_4_Ser)_2_ linker.

The cDNA encoding GUSh was cloned into the new *Not*I site at the 3′ end of the linker. Therefore, the *Not*I site present within the cDNA encoding GUSh was eliminated first. To this end, the cDNA encoding GUSh (Oshima *et al*, 1987) was recloned into pcDNA3 (Invitrogen, Groningen, The Netherlands). A primer, GUSh *Not*I mut, was designed with one silent mismatch within the sequence encoding the *Not*I site and overlying the *Kpn*I site just 5′ of the *Not*I site (Table 1). A PCR was performed using this primer and a primer that anneals at the BGH polyadenylation site of pcDNA3/GUSh. The PCR product was digested with *Kpn*I and *Sac*II. This fragment containing the mutated *Not*I site was used to replace the *Kpn*I/*Sac*II fragment in pcDNA3/GUSh.

The oligonucleotide pair GUSh *Not*I 5′ and GUSh *Not*I 3′ was then used to amplify the cDNA encoding mature GUSh (Table 1). Both primers contained a *Not*I site at the 5′ end. The PCR fragment was cut with *Not*I, and inserted into the *Not*I site of the vector containing C28 with (Gly_4_Ser)_2_ linker to obtain pC28-GUSh.

### Expression and purification of C28-GUSh fusion protein

To obtain stable transfectants, the pC28-GUSh was transfected into CHO cells by Lipofectamine Plus reagent (Life Technologies). Cells were grown in complete culture medium for 48 h. Transfected cells were selected by limiting dilution in medium containing 800 μg ml^−1^ zeocin (Invitrogen). Resistant clones were grown in medium supplemented with 400 μg ml^−1^ zeocin and screened for β-glucuronidase activity in culture medium as described below.

### β-Glucuronidase activity

Supernatants of stably transfected CHO clones were analysed for β-glucuronidase activity. To this end, 10 μl supernatant was suspended in 100 μl 1 mM 4-MuGlu in sodium acetate buffer (pH 4.2) with 0.1% BSA and incubated for 1 h at 37°C. The reaction was terminated by the addition of 1 ml 0.1 M glycine/NaOH pH 10.6 to 50 μl reaction mix. The fluorescent product, 4-methylumbelliferone, was measured using an excitation wavelength of 370 nm and an emission wavelength of 450 nm on the Fluorescence Spectrometer 3000 (Perkin Elmer). The stable CHO clone with the highest amounts of β-glucuronidase activity in the supernatant, CHO/C28-GUSh, was selected for further experiments.

### Western blot analysis

Western blot analysis was used to assess the size of the fusion protein. Supernatant from CHO/C28-GUSh (15 μl) was dissolved in sample buffer (Laemmli, 1970) with 2-mercaptoethanol and boiled at 95°C for 5 min. Samples were subjected to electrophoresis through a 10% sodium dodecyl sulphate-polyacrylamide gel under denaturing conditions. Protein bands were subsequently electroblotted onto PVDF protein membrane (Bio-Rad, Veenendaal, The Netherlands). Recombinant C28-GUSh fusion protein was detected using anti-myc antibody 9E10 (Chan *et al*, 1987), and horseradish peroxidase (HRP)-conjugated rabbit anti-mouse IgG (Dako, Glostrup, Denmark). Blots were developed with Lumilight Plus (Roche).

### FACS analysis

Binding of C28-GUSh to OVCAR-3 cells was determined by FACS analysis. OVCAR-3 cells were trypsinized for 5 min at 37°C, washed with DMEM, counted and resuspended in PBS. A total of 0.5×10^6^ cells were incubated with 50 μl supernatant of CHO/C28-GUSh or normal CHO cells on ice for 1 h and washed three times with PBS. Then, cells were incubated with the mouse anti-myc antibody 9E10 (Chan *et al*, 1987) in PBS/0.1% BSA, washed three times with PBS, and stained with fluorescein-conjugated rabbit anti-mouse IgG (Dako). Stained cells were fixed with 1% formaldehyde in PBS and analysed on a FACScan flow cytometer (Becton Dickinson, Erembodegem-Aalst, Belgium).

### Cell-associated* β-*glucuronidase activity

The C28-GUSh fusion protein in supernatant from CHO/C28-GUSh was incubated with OVCAR-3 cells for 1 h at 4°C to demonstrate cell-associated enzymatic activity. Specificity was determined by preincubation with the anti-Ep-CAM antibody 323/A3 (10 μg ml^−1^) to prevent binding of the fusion protein. Unbound material was separated from the cells by centrifugation. The cell pellet was washed with PBS containing 0.1% BSA and was suspended in 150 μl 1 mM 4-MuGlu in buffer (pH 4.2) for measurement of β-glucuronidase activity as described above. The reaction was stopped after 30 min and fluorescence was measured.

### Quality control of the fusion protein

In order to determine the binding and enzyme activity of the fusion protein as compared to the antibody and the enzyme alone, we transiently transfected COS-7 cells with pSTCF/C28, pcDNA3.1mycHis/GUSh or pC28GUSh. Cell lysates of transfected cells were analyzed on Western blot by anti-myc staining as described above. The intensity of all three protein bands was quantified using an imaging densitometer (Model GS-690, Biorad). Based on this semi-quantitative method, equal amounts of C28 and C28GUSh protein were used for FACS analysis on OVCAR-3 cells as described above. An enzyme activity assay was performed, as described above, with comparable amounts of GUSh and C28GUSh.

### *In vitro* antiproliferative effects

The *in vitro* antiproliferative effects of the prodrug DOX-GA3 on OVCAR-3 cells incubated with C28-GUSh fusion protein were determined as previously described (Haisma *et al*, 1998b). In a separate sample, preincubation with an excess of anti-Ep-CAM antibody 323/A3 (10 μg ml^−1^) was done to evaluate binding specificity of C28-GUSh. After 96 h the wells were incubated with cell proliferation reagent WST-1 (Roche) for 1 h at 37°C. The absorbance was measured at a wavelength of 450 nm. The antiproliferative effects were determined and expressed as IC_50_ values, the concentrations that give 50% growth inhibition compared with control cell growth.

### Bystander effect

CHO/C28-GUSh cells were plated together with Ep-CAM positive OVCAR-3 cells, or as a negative control with the Ep-CAM negative cell line U118, at a ratio of 1 : 10 in a six-well plate (2×10^5^ cells per well), and were grown for 72 h. Then, cells were washed three times with culture medium to remove unbound fusion protein and were incubated with 10 μM DOX-GA3 for 8 h. Cells were fixed with 3.7% paraformaldehyde and permeabilized with 0.2% Triton. Expression of C28-GUSh was detected by indirect immunofluorescence with mouse anti-myc antibody 9E10 (Chan *et al*, 1987) and fluorescein-conjugated rabbit anti-mouse secondary antibody (Dako) using confocal laser scan microscopy. Converted prodrug was seen as red autofluorescence of the nuclei, due to DNA-intercalating doxorubicin, with an emission maximum of 600 nm.

## RESULTS

### Construction of C28-GUSh fusion protein

**Figure 1:**
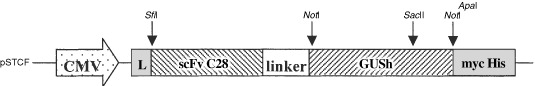
Schematic representation of the C28-GUSh expression cassette. The structural elements include the cytomegalovirus (CMV) promotor, IgG kappa leader sequence (L), and a C-terminal myc- and 6His-tag (mycHis) for easy detection and purification. The anti-Ep-CAM scFv C28 is inserted as an *Sfi*I/*Not*I fragment. The gene encoding GUSh is inserted as a *Not*I/*Not*I fragment, but not before the (Gly_4_Ser)_2_ linker is inserted in the *Not*I and *Apa*I restriction sites. The primers encoding the linker were designed in such a way that the *Not*I site at the 3′ end of the scFv disappeared upon downstream insertion, and a new one is introduced at the 3′ end of the linker.

The plasmid pC28-GUSh was constructed as shown in Figure 1

. The cDNA encoding the anti-Ep-CAM scFv C28 was isolated from a pHEN vector and inserted into the pSTCF vector (Arafat *et al*, 2000). The cDNA encoding GUSh was inserted in frame with the scFv separated by a 15-amino-acid linker segment containing (Gly_4_Ser)_2_ flanked on both sides by three alanine residues, encoded by the *Not*I sites. The signal peptide of GUSh was removed. The open reading frame encoded a recombinant protein, which after cleavage of the secretory signal sequence was composed of 913 amino acids with a predicted molecular mass of 102 kDa, including a myc- and 6His-tag at the C-terminus.

### Expression and characterisation of the fusion protein

**Figure 2:**
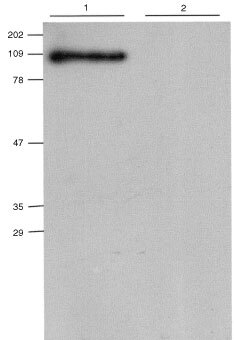
Western blot analysis of the C28-GUSh fusion protein. The molecular weight of C28-GUSh was determined by SDS–PAGE/Western blot using an anti-myc antibody 9E10 (Chan *et al*, 1987). The sizes of the molecular mass markers (Kaleidoscope prestained standards, Biorad) are indicated on the left. Lane 1: supernatant of the stable CHO/C28GUSh cell line. Lane 2: supernatant of untransfected CHO cells. The apparent mass of the full length C28-GUSh fusion protein is approximately 100 kDa, as expected.

A stable CHO cell line expressing C28-GUSh fusion protein was constructed. Clones were screened for β-glucuronidase activity in supernatant. The clone with the highest activity was chosen for further evaluation and was called CHO/C28-GUSh. Secreted fusion protein present in the supernatant of these cells was analysed by SDS–PAGE and Western blotting with an anti-myc antibody. The fusion protein monomers migrated with an apparent molecular weight of about 100 kDa (Figure 2

). The apparent molecular mass under non-denaturing conditions as determined by size exclusion chromatography was approximately 403 kDa (data not shown), which is in agreement with GUSh being active as a tetramer (Brot *et al*, 1978).

**Figure 3:**
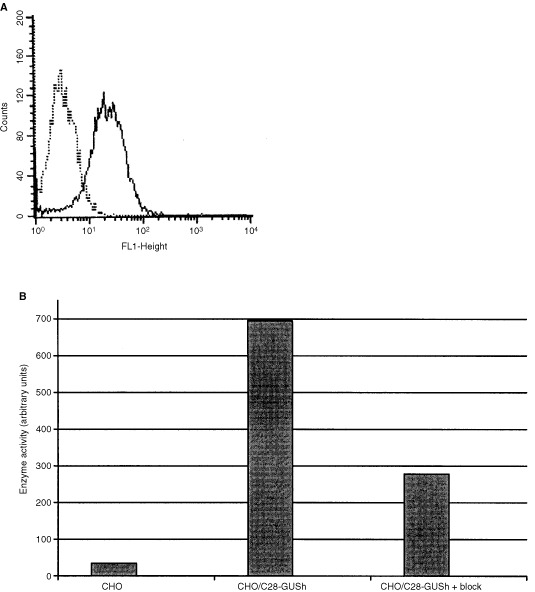
(**A**) Binding of the secreted C28-GUSh fusion protein to OVCAR-3 cells as measured by FACS. Ep-CAM positive OVCAR-3 cells were incubated with supernatant of the CHO/C28-GUSh cell line (solid line). OVCAR-3 cells exposed to supernatant of untransfected CHO cells served as a negative control (dotted line). Binding was visualised with mouse anti-myc antibody and fluorescein-conjugated rabbit anti-mouse IgG. (**B**) OVCAR-3 cells incubated with supernatant derived from CHO/C28-GUSh (lane 2) or untransfected CHO cells (lane 1) were analysed for cell-associated enzyme activity. Binding specificity was tested by incubating OVCAR-3 cells with supernatant of CHO/C28-GUSh together with an excess of the anti-Ep-CAM antibody, 323/A3 (lane 3). Unbound material was separated from the cells by centrifugation. The pelleted cells were incubated with 1 mM 4-MuGlu as a substrate and fluorescence was measured after 30 min.

The binding of the fusion protein in CHO/C28-GUSh supernatant to Ep-CAM expressing OVCAR-3 cells was demonstrated by FACS analysis (Figure 3A

). Supernatant from untransfected CHO cells served as negative control. Binding of functional fusion protein was confirmed by an assay for cell-associated enzyme activity (Figure 3B). There was indeed β-glucuronidase activity associated with cells incubated with CHO/C28-GUSh supernatant, and not with cells incubated with supernatant from untransfected CHO cells. This activity was specifically bound to Ep-CAM, because incubation with an excess of the anti-Ep-CAM antibody 323/A3 together with CHO/C28-GUSh supernatant resulted in a significantly reduced level of β-glucuronidase activity bound to OVCAR-3 cells.

In order to determine the binding and enzyme activity of the fusion protein as compared to the antibody and the enzyme alone, we performed, respectively, a FACS analysis and an enzyme activity assay with comparable amounts of protein. The binding of C28GUSh to OVCAR-3 cells was at least 50% of that from a comparable amount of C28 alone. Furthermore, the enzyme activity of C28GUSh was also at least 50% of the activity of GUSh alone (data not shown).

### Prodrug activation and antiproliferative effects

OVCAR-3 cells were used to determine the specific activation of the prodrug DOX-GA3 by C28-GUSh to doxorubicin.

Under the experimental conditions used, the IC_50_ value for doxorubicin in OVCAR-3 cells was 5 μM, which confirmed the 2.5 μM value calculated in a previous experiment (Houba *et al*, 2001a). This relatively high IC_50_ value could probably be explained by the use of U-bottom wells, in which the cells do not grow as a monolayer, but more like a spheroid. Therefore, not all cells are in direct contact with the drug. This might cause the relatively high IC_50_ value of doxorubicin in our system.

**Figure 4:**
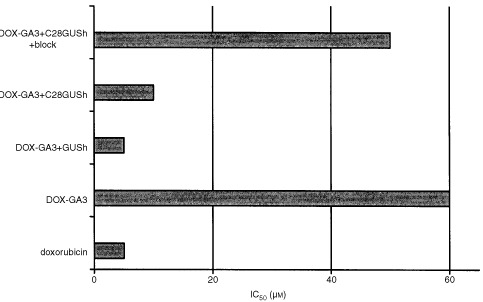
Antiproliferative effects of doxorubicin, DOX-GA3, and activated DOX-GA3. Cells were incubated with supernatant of CHO/C28-GUSh without or with preincubation with an anti-Ep-CAM antibody as blocking agent or PBS. Cells were then seeded in 96-well plates and exposed to drug or prodrug in triplo. As a control, cells were grown in the presence of 0.25 U ml^−1^ GUSh and prodrug. After 72 h, cell growth was measured with a protein dye, WST-1, and the IC_50_ values were determined.

The prodrug alone was relatively non-toxic with an IC_50_ value of approximately 60 μM. To determine the effects of prodrug completely hydrolysed by the enzyme, an excess of β-glucuronidase (0.25 U ml^−1^) was used. Indeed, the IC_50_ value was the same as the IC_50_ value for doxorubicin, namely about 5 μM. In other words, the prodrug was completely converted to doxorubicin. Similarly, preincubation of OVCAR-3 cells with C28-GUSh increased the antiproliferative effects of the prodrug as the IC_50_ value decreased from 60 to 10 μM as a result from activation (Figure 4

). This effect could be inhibited almost completely by preincubation with the anti-Ep-CAM antibody 323/A3.

In order to assess a potential bystander effect due to prodrug conversion by secreted and targeted GUSh, we mixed CHO/C28-GUSh with OVCAR-3 or U118 cells, representing, respectively, Ep-CAM positive and Ep-CAM negative cells. For this purpose, we made use of the strong autofluorescence of doxorubicin. DOX-GA3 is hydrophilic and cannot pass cell membranes. After cleavage of the prodrug, the generated hydrophobic doxorubicin can pass cell membranes and can be detected as red nuclear fluorescence within the cells. This experiment was carried out with 10% C28-GUSh expressing cells and 90% OVCAR-3 or U118 cells. Three days after plating, cells were washed to discard unbound fusion protein and prodrug was added for 8 h. The presence of C28-GUSh was visualised by indirect immunohistochemistry for myc-tagged protein.

**Figure 5:**
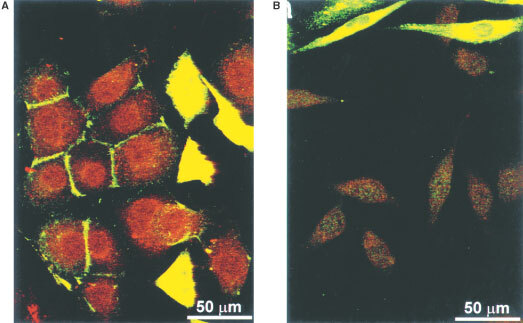
Tumour-specific prodrug conversion by C28GUSh. CHO/C28-GUSh cells were plated after mixture with Ep-CAM positive OVCAR-3 cells (**A**) or Ep-CAM negative U118 cells (**B**), as a negative control, at a ratio of 1 : 10. After 3 days of growth, medium was aspirated and cells were washed and subsequently incubated with medium containing 10 μM DOX-GA3 for 8 h. Thereafter, cells were fixed and C28-GUSh expressing cells and cells which had bound C28-GUSh were detected by indirect immunofluorescence using an anti-myc antibody and a fluorescein conjugated secondary antibody (green staining). Prodrug conversion is visualised as the red nuclear staining of doxorubicin within the cells.

OVCAR-3 cells showed membrane staining by bound C28-GUSh (Figure 5A

, green fluorescence), whereas the membranes of the Ep-CAM negative U118 cells mixed with CHO/C28-GUSh cells did not contain staining (Figure 5B). All nuclei of OVCAR-3 cells mixed with CHO/C28-GUSh cells were strongly positive for doxorubicin (Figure 5A, red fluorescence). This is the result of prodrug conversion by membrane-bound fusion protein, as no nuclei stained positive for doxorubicin in the wells containing U118 cells mixed with CHO/C28-GUSh cells (Figure 5B). Furthermore, untransfected CHO cells mixed with OVCAR-3 cells did also not show any membrane staining and prodrug conversion (data not shown). The cytosol of C28-GUSh expressing CHO cells stained strongly positive for myc-tagged fusion protein. Due to the high levels of C28-GUSh in CHO cells, cytosolic fluorescence detected with the anti-myc antibody interfered with doxorubicin autofluorescence resulting in yellow staining instead of green.

## DISCUSSION

We have constructed an expression plasmid for the production of a single-chain antibody-enzyme fusion protein composed of the human anti-Ep-CAM scFv antibody C28 and the human lysosomal enzyme GUS. The C28-GUSh fusion protein was expressed by eukaryotic CHO cells containing the expression construct and secreted into the supernatant of these cells. The fusion protein was present as a tetramer in the supernatant. The secreted fusion protein retained at least 50% of enzymatic activity, bound specifically to Ep-CAM-expressing cells, and was able to convert the glucuronide-prodrug DOX-GA3. We expect that the activation rate for the conversion of prodrug to drug is comparable to the activation rate by the enzyme alone, as we have previously shown for a similar fusion protein consisting of a murine scFv antibody and β-glucuronidase (Haisma *et al*, 1998b). The C28-GUSh fusion protein is fully functional and could therefore be useful for selective activation of a relatively non-toxic prodrug at the site of the tumour.

A fully human fusion protein is less likely to elicit an immune response in patients than a fusion protein containing non-human peptides. The immune response in patients has been reported to limit ADEPT to only one cycle of treatment (Sharma *et al*, 1992). In that particular study, human anti-mouse antibodies (HAMA) and human anti-enzyme antibodies were detected in all patients treated with a mouse monoclonal antibody conjugated to the bacterial enzyme carboxypeptidase G2. The immunosuppressive agents, cyclosporin A or deoxyspergualin, can delay the host immune response allowing two or three cycles of treatment to be given (Dhingra *et al*, 1995; Bagshawe and Sharma, 1996). Cyclosporin A, however, leads to additional toxicity. Development of HAMA and human anti-enzyme antibodies might be prevented by using a human antibody and a human enzyme.

Previously, the functional characterisation of a fusion protein consisting of the humanized fragment of an anti-CEA monoclonal antibody and GUSh expressed in BHK cells has been reported (Bosslet *et al*, 1992). The use of a humanised antibody fragment in a fusion protein is not ideal. Although humanised antibodies are less immunogenic than murine antibodies, anti-idiotypic antibodies can develop as was shown in patients as well as in Rhesus monkeys (Reimann *et al*, 1997; Richards *et al*, 1999). This may be due to the variable regions of humanised antibodies being still of murine origin.

As an alternative to ADEPT, gene-directed enzyme prodrug therapy (GDEPT) is currently under study. In the GDEPT approach, the gene encoding the enzyme is delivered selectively to tumour cells. The advantage of GDEPT is that the enzyme is continuously produced by the transduced cells, resulting in accumulation of the enzyme in the tumour. It has recently been shown that GDEPT using tumour cells expressing a secreted form of GUSh is effective in induction of tumour cell killing *in vitro* and *in vivo* (Weyel *et al*, 2000). The presence of approximately 7% transduced cells was sufficient to obtain a therapeutic effect in a mouse model. A potential disadvantage is, however, that untargeted GUSh may leak from the tumour, resulting in conversion of prodrug outside of the tumour area. In fact, leakage of GUS from the site of production has been demonstrated (Stein *et al*, 1999). The aim of that study was correction of mucopolysaccharidosis type VII, a lysosomal storage disease caused by a deficiency in functional GUS. For systemic correction of GUS deficiency, diffusion of the enzyme from the liver is necessary. Indeed, after intravenous delivery of an adenovirus expressing GUS resulting in transduction of mainly liver cells, the investigators found corrective levels of GUS activity in blood and uptake at sites that are poorly transduced, such as kidneys and lungs.

In GDEPT, diffusion of the enzyme from the tumour should be prevented at all costs, as it will negatively affect tumour selectivity. The solution to this problem could be secretion of a targeted form or a surface tethered form of GUSh by transduced tumour cells, as it might result in retention of the enzyme within the tumour. Recently, a surface tethered form of GUSh has been constructed by fusion of the transmembrane domain of the human PDGF receptor to a C-terminally truncated form of GUSh (Heine *et al*, 2001). This GDEPT system has been shown to produce a potent anti-tumour effect *in vivo*. We hypothesise that secretion of a targeted form of GUSh may result in a more pronounced bystander effect, as the enzyme will diffuse within the tumour and bind to neighbouring antigen-positive cells. The C28-GUSh construct is a possible candidate for this purpose. In view of this, we have shown *in vitro* that 10% of C28-GUSh expressing cells suffice to achieve uptake of doxorubicin into all cells in the population. Moreover, the secreted fusion protein C28-GUSh could bind specifically to untransduced, Ep-CAM positive cells. This is very important for future *in vivo* application, as not all cells in the tumour will be transduced with the fusion construct.

In summary, we have engineered a fully human and functional fusion protein for future application in selective chemotherapy. This construct could be produced on a large-scale for ADEPT. In addition, it could be used for cancer gene therapy. In the latter case, the fusion protein would be produced locally within the tumour by infected tumour cells, and bind to the relevant antigen. The secretion of a targeted enzyme within the tumour should ensure a high therapeutic index for a clinically important cytotoxic drug when activated specifically at the site of the tumour.
